# Labdane Diterpenes from the Fruits of *Sinopodophyllum emodi*

**DOI:** 10.3390/molecules21040434

**Published:** 2016-03-31

**Authors:** Yan-Jun Sun, Mei-Ling Gao, Yan-Li Zhang, Jun-Min Wang, Ya Wu, Yu Wang, Tao Liu

**Affiliations:** 1 Collaborative Innovation Center for Respiratory Disease Diagnosis and Treatment & Chinese Medicine Development of Henan Province, Henan University of Traditional Chinese Medicine, Zhengzhou 450046, Henan, China; gaoxiaomei6266@126.com (M.-L.G.); zyl2013hnzy@163.com (Y.-L.Z.); wjmhnzz@163.com (J.-M.W.); wuya0723@126.com (Y.W.); 2 School of Pharmacy, Henan University of Traditional Chinese Medicine, Zhengzhou 450046, Henan, China; 3 School of Pharmacy, China Medical University, Shenyang 110122, Liaoning, China; xiaowangyu21@163.com

**Keywords:** *Sinopodophyllum emodi*, labdane diterpene, cytotoxic activity

## Abstract

Two new labdane diterpenes, sinoditerpene A (**1**) and B (**2**), were isolated from the fruits of *Sinopodophyllum emodi*, along with two known analogues **3** and **4**. Their structures were established on the basis of extensive spectroscopic analysis. The isolation of compounds **1**–**4** represents the first report of diterpenes from the genus *Sinopodophyllum*. The cytotoxic activities of all isolated compounds were evaluated in comparison with 5-fluorouracil against the MCF-7 and HepG2 cell lines, towards which **3** showed more potent cytotoxicity.

## 1. Introduction

**Figure 1:**
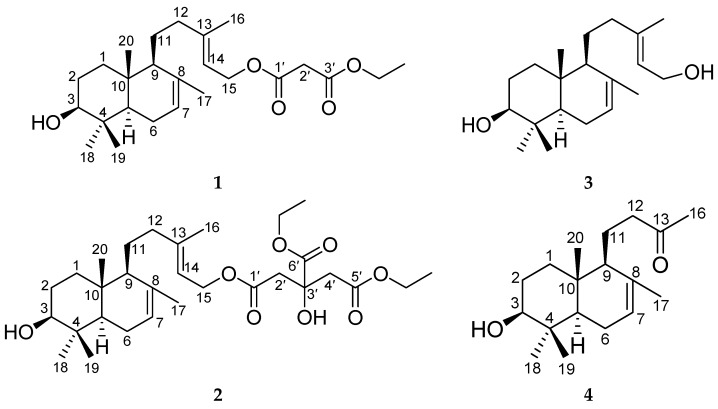
The chemical structures of compounds **1**–**4** from *S. emodi*.

*Sinopodophyllum emodi* is an important medicinal plant that has been described in the Chinese Pharmacopoeia and Tibetan medicine [1]. Its dried roots and rhizomes (called Taoerqi in Chinese) are frequently used for the treatment of certain cancers, various verrucosis [2], constipation, verminosis [3], rheumatoid pain [4], and pyogenic skin tissue infections [5]. The dried ripe fruits (called “Xiaoyelian” in Chinese) are clinically applied to the treatment of amenorrhea, dead fetus, and placental retention [1]. Previous phytochemical and pharmacological investigations revealed that *S. emodi* is particularly rich in aryltetralin lactone lignans and prenylated flavonoids, and has attracted wide attention due to their cytotoxic properties [1,2,3,6,7,8,9]. In our search for cytotoxic natural products, we previously reported the isolation, identification and cytotoxic activity of aryltetralin lactone and tetrahydrofuranoid lignans, and prenylated flavonoids from *S. emodi* [10,11,12]. In a further examination of the fruits of this plant, two new labdane diterpenes, sinoditerpene A (**1**) and B (**2**), were obtained together with two known analogues **3** and **4** (Figure 1). For the first time, the NMR signals for compound **4** were completely assigned by 2D NMR spectra (^1^H-^1^H COSY, HSQC, HMBC, NOESY). Details of the isolation, structure elucidation, and cytotoxicity of all isolated compounds against MCF-7 and HepG2 cell lines are described here.

## 2. Results and Discussion

The EtOH extract of the fruits of *Sinopodophyllum* *emodi* was partitioned between PE, CH_2_Cl_2_, EtOAc, *n*-BuOH and water, respectively. The CH_2_Cl_2_ layer was fractionated and purified by repeated column chromatography, allowing the isolation of four labdane diterpenes **1**–**4**. By comparing their physical and spectroscopic data (^1^H-NMR, ^13^C-NMR, ^1^H-^1^H COSY, HSQC, HMBC, and NOESY in Supplementary Materials) with literature values [13], the two known metabolites were identified as labda-7,13-diene-3,15-diol (**3**) and 14,15-dinor-3β-hydroxy-7-labden-13-one (**4**). The chemical structures of two new labdane diterpenes were determined on the basis of spectroscopic evidences (^1^H-NMR, ^13^C-NMR, ^1^H-^1^H COSY, HSQC, HMBC, DEPT, and NOESY in Supplementary Materials), and their absolute configurations were elucidated by [α]_D_ and NOESY analysis.

**Figure 2:**
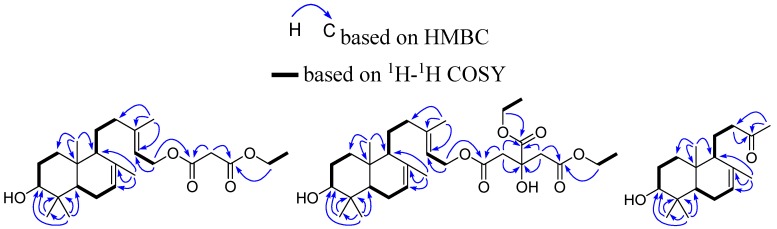
Key ^1^H-^1^H COSY, HMBC correlations of compounds **1**–**3**.

Compound **1** was obtained as a sticky oil and possessed a molecular formula C_25_H_40_O_5_ with six degrees of unsaturation, as revealed from its HR-ESI-MS analysis (*m*/*z* 443.2771 [M + Na]^+^, calcd 443.2773). The IR spectrum displayed the presence of carbonyl (1736 cm^−1^) and hydroxy (3464 cm^−1^) groups. The ^13^C-NMR and DEPT spectra showed twenty-five carbon signals, including six quaternary carbons, six methyls, eight methylenes and five methines. The ^1^H- and ^13^C-NMR spectra (Table 1 and Table 2) revealed the presence of malonic acid monoethyl ester [14] and a labdane skeleton [13,15,16]. One oxygenated methylene [δ_H_ 4.18 (2H, q, *J* = 7.2 Hz), δ_C_ 62.3], one methyl [δ_H_ 1.26 (3H, t, *J* = 7.2 Hz), δ_C_ 14.1], one methylene [δ_H_ 3.22 (2H, s), δ_C_ 41.7], and two ester carbonyls [δ_C_ 166.7, 166.6] were observed, suggesting the presence of malonic acid monoethyl ester [14]. Two ester carbonyl [δ_C_ 166.7, 166.6], and four olefinic carbons δ_C_ 117.9, 122.3, 135.0, 143.3 accounted for four out of the six degrees of unsaturation, and the remaining two indicated that compound **1** was bicyclic. Five methyls [δ_H_ 0.74 (3H, s), 0.83 (3H, s), 0.95 (3H, s), 1.67 (3H, br.s), 1.70 (3H, br.s), δ_C_ 13.6, 15.0, 16.6, 21.9, 27.9], six methylenes [one oxygenated δ_H_ 4.64 (2H, d, *J* = 7.2 Hz), δ_C_ 61.5], five methine [one oxygenated δ_H_ 3.22 (1H, dd, *J* = 11.2, 4.5 Hz), δ_C_ 79.1; two olefinic δ_H_ 5.38 (1H, br.s), 5.32 (1H, t, *J* = 7.2 Hz)], and four olefinic carbons [δ_C_ 117.9, 122.3, 135.0, 143.3], suggested that compound **1** possessed a 3,15-dihydroxy-7,13-labdadien skeleton [13]. The HMBC correlation (Figure 2) between the ester carbonyl δ_C_ 166.7 (C-1’) and the methylene δ_H_ 4.64 (2H, d, *J* = 7.2 Hz, H-15), indicated that the 15-OH was esterified by malonic acid monoethyl ester.

**Figure 3:**
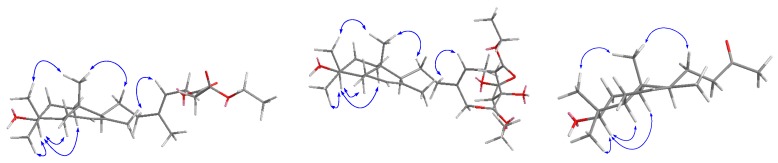
Key NOESY correlations of compounds **1**–**3**.

The relative configuration of the ring substituents of compound **1** was determined by analyzing the NOESY spectrum (Figure 3). The NOESY correlations from H-3 to Me-18, H-5, and H-1α indicated that they were cofacial and were assigned α-orientation. The NOESY cross peak of Me-19/Me-20 and Me-20/H-11 showed that these protons were β-oriented. Additionally, the stereochemistry of the side-chain double bond was established as *E* by virtue of the Me-16 chemical shifts (δ_H_ 1.70; δ_C_ 16.6) [13]. This was also supported by the cross peak of H-12/H-14 in the NOESY spectrum. The absolute configuration is the same as (−)-labda-7,13-diene-3,15-diol [17,18], which was determined by a microhydrolysis method and comparison of [α]_D_ values. Thus, compound **1** was established as 3β-hydroxy-15-(3’-ethoxy-3’-oxopropionyloxy)-7,13*E*-labdadiene, and named sinoditerpene A.

Compound **2** was obtained as a sticky oil and possessed a molecular formula C_30_H_48_O_8_ with seven degrees of unsaturation, as revealed from its HR-ESI-MS analysis (*m*/*z* 559.3249 [M + Na]^+^, calcd 559.3247). The IR spectrum displayed the presence of carbonyl (1734, 1718 cm^−1^) and hydroxyl (3465 cm^−1^) groups. Its ^1^H- and ^13^C-NMR data (Table 1 and Table 2) were quite similar to those of compound **1**, except that citric acid-1,2-diethyl ester was observed instead of the malonic acid monoethyl ester of compound **1**. Two hydroxymethyls [δ_H_ 4.12 (2H, q, *J* = 7.2 Hz), 4.26 (2H, q, *J* = 7.2 Hz), δ_C_ 61.0, 62.3], two methyls [δ_H_ 1.26 (3H, t, *J* = 7.2 Hz), 1.28 (3H, t, *J* = 7.2 Hz), δ_C_ 14.0, 14.1], two methylenes [δ_H_ 2.76 (1H, d, *J* = 15.6 Hz), 2.87 (1H, d, *J* = 15.6 Hz), 2.76 (1H, d, *J* = 15.6 Hz), 2.87 (1H, d, *J* = 15.6 Hz), δ_C_ 43.2, 43.3], and three ester carbonyls [δ_C_ 169.76, 169.80, 173.4] were observed, suggesting the presence of citric acid-1,2-diethyl ester [19]. The HMBC correlation between the ester carbonyl δ_C_ 169.80 (C-1’) and the methylene δ_H_ 4.58 (2H, d, *J* = 7.5 Hz, H-15), indicated that the 15-OH was esterified by citric acid-1,2-diethyl ester. Thus, compound **2** was established as 3β-hydroxy-15-[(3’-hydroxy-3’,4’-bis(ethoxycarbonyl)-butyroxy)]-7,13*E*-labdadiene, and named sinoditerpene B.

Compound **4** was obtained as a sticky oil and possessed a molecular formula C_18_H_30_O_2_ with four degrees of unsaturation, as revealed from its HR-ESI-MS analysis (*m*/*z* 301.2148 [M + Na]^+^, calcd 301.2143). The IR spectrum displayed the presence of carbonyl (1714 cm^−1^) and hydroxy (3415 cm^−1^) groups. Its ^1^H- and ^13^C-NMR (Table 1 and Table 2) were similar to those of compound **3**, except that a carbonyl group δ_C_ 208.7 were observed instead of two olefinic carbons δ_C_ 140.0, 123.5, and one oxygenated methylene δ_C_ 59.4 in **1**. Due to apparent differences of carbon signal number in **4** (20 carbons) and **3** (18 carbons), it was reasonably assumed that **4** was a dinor-derivative of **3**. The HMBC correlation between the carbonyl group δ_C_ 208.7 (C-13) and the methyl δ_H_ 2.11 (3H, s, H-16), the methylene δ_H_ 2.40 (1H, m, H-12), 2.63 (1H, m, H-12), in combination with ^1^H-^1^H COSY cross peak of H-11 with H-12 and H-9, indicated that 3-oxobutyl was linked to C-9. On the basis of the above evidences and related literature [13], compound **4** was established as 14,15-dinor-3β-hydroxy-7-labden-13-one.

Chinese herbal medicines produce a wide variety of secondary metabolites, which can be exploited as potential anticancer agents. Previous chemical investigations on *S. emodi* revealed the presence of aryltetralin lactone and tetrahydrofuranoid lignans [2,3,7,8,10], flavonoids [1,6,9,11,20], steroids [21], and phenolics [22]. Cytotoxic activities of some isolated *S. emodi* constituents have been shown in various cancer cell lines. In the HeLa and KB cell lines, deoxypodphyllotoxin was about 579 and 1123 times more toxic than etoposide, respectively [10]. Sinolignan C displayed cytotoxicity against the KB cell line and was more cytotoxic than etoposide [12]. 3-Methoxyquercetin showed cytotoxicity against the MCF-7 and HepG2 cell lines, with IC_50_ values of 3.14 and 2.08 μM, respectively [11]. All of the isolated labdane diterpenoids were evaluated for cytotoxic activities against the MCF-7 and HepG2 cell lines (Table 3). Compound **3** was more cytotoxic than 5-fluorouracil, whereas compounds **1**, **2** and **4** displayed no cytotoxicity against MCF-7 and HepG2 cell lines. Compounds **1**–**4** have the same structural skeleton, so the variation in cytotoxicity between them indicates a free hydroxyl group at C-15 was structurally required for the cytotoxity against the MCF-7 and HepG2 cells lines. Esterification at C-15 drastically reduced the cytotoxic activity of the parent compound **3**. These results revealed the potential of compound **3** as an ideal antitumor lead compound.

## 3. Experimental Section

### 3.1. General Procedures

The IR spectra were measured on a Tensor 27 Fourier transform infrared (FTIR) spectrometer (Bruker Optics, Ettlingen, Germany) as KBr discs. The 1D and 2D NMR spectra were recorded on an AC (E)-500 spectrometer(Bruker Biospin, Fallanden, Switzerland) using TMS as an internal standard. HR-ESI-MS was determined on a microTOF-Q instrument (Bruker Daltonics, Billerica, MA, USA). Optical rotations were measured on an Autopol IV Digital Polarimeter (Rudolph Research Analytical, Hackettstoun, NJ, USA). The chromatographic silica gel (200–300 mesh) was obtained from Qingdao Ocean Chemical Factory (Qingdao, China). ODS (50 µm) was produced from YMC Co. Ltd. (Kyoto, Japan). Sephadex LH-20 was produced by Amersham Pharmacia Biotech (Uppsala, Sweden). Chemical reagents for isolation were of analytical grade and purchased from Tianjin Siyou Co., Ltd. (Tianjin, China). Biological reagents were from Sigma Company (St. Louis, MO, USA). Human heptocellular (HepG2) and breast (MCF-7) cell lines were from Institute of Materia Medica, Chinese Academy of Medical Sciences and Peking Union Medical College (Beijing, China).

### 3.2. Plant Material

The plant material was collected from Deqin, Yunnan Province, China, in September 2013, and identified by Prof. Cheng-Ming Dong as the fruits of *S. emodi*, according to the Chinese Traditional Medicine Dictionary [23]. A voucher specimen (SE 20130929) was deposited at the School of Pharmacy, Henan University of Traditional Chinese Medicine.

### 3.3. Extraction and Isolation

The dried fruits of *S. emodi* were ground into a power (9.1 kg), and refluxed with 95% EtOH(3 × 20 L). The filtrate was concentrated under reduced pressure to yield a dark brown residue (1.6 kg). The residue was suspended in water (3.2 L) and partitioned with petroleum ether (PE, 3.2 L × 3), CH_2_Cl_2_ (3.2 L × 3), EtOAc (3.2 L × 3), and *n*-BuOH (3.2 L × 3), successively. The CH_2_Cl_2_ extract (400.05 g) was fractionated using silica gel column chromatography (CC, 20 × 140 cm) with a gradient of PE (60–90 °C)–acetone. The fractions were combined into eleven main fractions C1–11 based on TLC results. Fraction C3 (5.02 g) was chromatographed over open ODS (2.5 × 45 cm), eluted by methanol–H_2_O (20: 80 to 85: 15) to yield subfractions C–3–1~C–3–3. Subfraction C–3–3 (0.95 g) was further submitted to silica gel CC (1.0 × 25 cm) eluted by PE–acetone (100: 20) to give **1** (7.8 mg) and **2** (4.6 mg). Fraction C4 (6.95 g) was subjected to Sephadex LH-20 CC (2.0 × 90 cm) eluted by methanol to yield subfractions C4–1 and C4–2. Subfractions C4–1 (1.74 g) was separated by open ODS (2.0 × 40 cm) eluted by methanol–H_2_O (40: 60 to 80: 20) to give **3** (20.8 mg) and **4** (5.2 mg).

### 3.4. Spectroscopic and Physical Data

*Sinoditerpene A* (**1**). Sticky oil; ${\left[\alpha \right]}_{D}^{25}$ –12.4 (*c* 0.14, CHCl_3_); IR (KBr)ν_max_ 3464, 2967, 2930, 2855, 1737 cm^−1^; HR-ESI-MS (positive): *m*/*z* 443.2771 [M + Na]^+^ (calcd for C_25_H_40_O_5_Na, 443.2773); NMR data (CDCl_3_), see Table 1 and Table 2.

*Sinoditerpene B* (**2**). Sticky oil; ${\left[\alpha \right]}_{D}^{25}$ –9.5 (*c* 0.16, CHCl_3_); IR (KBr)ν_max_ 3456, 2956, 2924, 2853, 1734, 1718; HR-ESI-MS (positive): *m*/*z* 559.3249 [M + Na]^+^ (calcd for C_30_H_48_O_8_Na, 559.3247); NMR data (CDCl_3_), see Table 1 and Table 2.

### 3.5. Alkali Hydrolysis

Compound **1** (6 mg) was refluxed with 4N alcoholic KOH (5 mL) for 1 h. The resulting liquid was neutralized by 1N HCI until pH 7, and concentrated under reduced pressure, and then extracted with CH_2_Cl_2_ (10 mL × 3) after 10 mL water was added. The organic phase was washed with water, dried over Na_2_SO_4_ and concentrated *in vacuo*. The residue was chromatographed on silica gel (PE–acetone 100:7–100:20) to obtain the hydrolyzed target compound.

### 3.6. Cytotoxicity Asssay

Tumor cells were maintained in RPMI-1640 medium containing 10% heat-inactivated fetal bovine serum, penicillin (100 units/mL), streptomycin (100 μg/mL) under humidified air with 5% CO_2_ at 37 °C. Exponentially growing cells were seeded into 96-well tissue culture-treated plates and precultured for 1 day. The cytotoxic activities of isolated compounds were tested against MCF-7 and HepG2 cell lines, using an established MTT assay protocol [10]. 5-fluorouracil was used as the positive control.

## 4. Conclusions

Further phytochemical studies on *S*. *emodi* resulted in the isolation of two new labdane diterpenes **1** and **2** and two known analogues **3** and **4**. All the labdane diterpenoids contained 7(8) and 13(14) double bonds. Isolation of diterpenoids from *S. emodi* is reported here for the first time, which also enriches our knowledge about the chemical diversity of this plant. Compound **1** is the first reported example of a labdane diterpene with 7(8) and 13(14) double bonds exhibiting cytotoxicity in the MCF-7 and HepG2 cell lines. As an antitumor lead compound, further investigations are necessary to explore the structure-activity relationship and lead optimization. Our research demonstrated that the fruits of *S. emodi* have chemopreventive potential as a herbal medicine and its labdane diterpenes are partly responsible for the observed potency. In addition, these results will broaden the application field of *S. emodi*.

## Figures and Tables

**Table 1:** ^1^H-NMR data (500 MHz, δ in ppm, *J* in Hz) of compounds **1**–**4** in CDCl_3_.

No.	1	2	3	4
1	1.07 (1H, td, 13.2, 4.2)	1.06 (1H, td, 13.1, 4.5)	1.06 (1H, td, 13.2, 4.4)	1.10 (1H, td, 13.3, 4.0)
	1.84 (1H, dt, 13.2, 3.4)	1.84 (1H, dt, 13.1, 3.4)	1.84 (1H, dt, 13.2, 3.4)	1.91 (1H, dt, 13.3, 3.6)
2	1.60 (2H, m)	1.60 (2H, m)	1.60 (2H, m)	1.60 (2H, m)
3	3.22 (1H, dd, 11.2, 4.5)	3.22 (1H, dd, 11.0, 4.8)	3.20 (1H, dd, 11.2, 4.5)	3.21 (1H, dd, 11.4, 4.4)
5	1.16 (1H, dd, 10.9, 6.1)	1.17 (1H, dd, 10.9, 6.1)	1.15 (1H, dd, 10.9, 6.1)	1.15 (1H, dd, 10.9, 6.1)
6	1.95 (2H, m)	1.95 (2H, m)	1.95 (2H, m)	1.95 (2H, m)
7	5.38 (1H, br.s)	5.38 (1H, br.s)	5.37 (1H, br.s)	5.39 (1H, br.s)
9	1.57 (1H, m)	1.58 (1H, m)	1.58 (1H, m)	1.57 (1H, m)
11	1.47 (1H, m)	1.50 (1H, m)	1.50 (1H, m)	1.78 (1H, m)
	1.25 (1H, m)	1.25 (1H, m)	1.28 (1H, m)	1.45 (1H, m)
12	1.95 (1H, m)	1.95 (1H, m)	1.95 (1H, m)	2.40 (1H, m)
	2.22 (1H, m)	2.20 (1H, m)	2.20 (1H, m)	2.63 (1H, m)
14	5.32 (1H, t, 7.2)	5.32 (1H, t, 7.5)	5.40 (1H, t, 7.0)	
15	4.64 (2H, d, 7.2)	4.58 (2H, d, 7.5)	4.13 (2H, d, 7.0)	
16	1.70 (3H, s)	1.68 (3H, s)	1.67 (3H, s)	2.11 (3H, s)
17	1.67 (3H, s)	1.68 (3H, s)	1.66 (3H, s)	1.64 (3H, s)
18	0.95 (3H, s)	0.95 (3H, s)	0.94 (3H, s)	0.94 (3H, s)
19	0.83 (3H, s)	0.83 (3H, s)	0.83 (3H, s)	0.83 (3H, s)
20	0.74 (3H, s)	0.73 (3H, s)	0.73 (3H, s)	0.76 (3H, s)
2′	3.22 (2H, s)	2.76 (1H, d, 15.6)		
		2.87 (1H, d, 15.6)		
4′		2.76 (1H, d, 15.6)		
		2.87 (1H, d, 15.6)		
CH_2_	4.18 (2H, q, 7.2)	4.12 (2H, q, 7.2)		
CH_3_	1.26 (3H, t, 7.2)	1.28 (3H, t, 7.2)		
CH_2_		4.26 (2H, q, 7.2)		
CH_3_		1.26 (3H, t, 7.2)		

**Table 2:** ^13^C-NMR data (125 MHz, δ in ppm) of compounds **1**–**4** in CDCl_3_.

No.	1	2	3	4	No.	1	2	3	4
1	37.2	37.2	37.1	37.4	16	16.6	16.6	16.5	30.0
2	27.3	27.4	27.3	27.4	17	21.9	21.9	21.9	22.0
3	79.1	79.1	79.1	79.1	18	27.9	27.9	27.8	27.9
4	38.6	38.6	38.6	38.7	19	15.0	15.0	15.0	15.1
5	49.5	49.5	49.5	49.5	20	13.6	13.6	13.6	13.6
6	23.4	23.4	23.4	23.4	1′	166.7	169.80		
7	122.3	122.3	122.1	122.9	2′	41.7	43.2		
8	135.0	135.0	135.1	134.3	3′	166.6	73.2		
9	54.3	54.2	54.3	54.3	4′		43.3		
10	36.6	36.6	36.6	36.7	5′		169.76		
11	25.5	25.3	25.5	20.8	6′		173.4		
12	41.9	41.8	41.9	45.7	CH_2_	62.3	62.3		
13	143.3	143.2	140.0	208.7	CH_3_	14.1	14.0		
14	117.9	118.0	123.5		CH_2_		61.0		
15	61.5	61.8	59.4		CH_3_		14.1		

**Table 3:** Cytotoxicities of compounds **1**–**4** against MCF-7 and HepG2 cell lines (IC_50_, μM).

Compound	MCF-7	HepG2	Compound	MCF-7	HepG2
**1**	74.6 ± 5.5	63.5 ± 6.2	**3**	5.73 ± 0.46	3.85 ± 0.29
**2**	88.3 ± 7.1	75.2 ± 6.8	**4**	50.2 ± 5.0	39.1 ± 4.7
5-Fluorouracil	6.74 ± 0.52	5.18 ± 0.40			

## Supplementary Materials

Supplementary materials can be accessed at: http://www.mdpi.com/1420-3049/21/4/434/s1.
