# Supplementary material for: Labdane Diterpenes from the Fruits of Sinopodophyllum emodi
Source: Molecules. 2016 Mar 31;21(4):434. doi: 10.3390/molecules21040434 (PMC6272936; doi:10.3390/molecules21040434)
Supplement: Supplementary file 1 [file molecules-21-00434-s001.pdf]

Supplementary Materials: Labdane Diterpenes from the Fruits of *Sinopodophyllum emodi*

Yan-Jun Sun, Mei-Ling Gao, Yan-Li Zhang, Jun-Min Wang, Ya Wu, Yu Wang and Tao Liu

List of Supplementary Materials

The 1D and 2D-NMR spectra of compound **1**....... 2
The 1D and 2D-NMR spectra of compound **2**....... 6
The 1D and 2D-NMR spectra of compound **3**....... 10
The 1D and 2D-NMR spectra of compound **4**....... 13

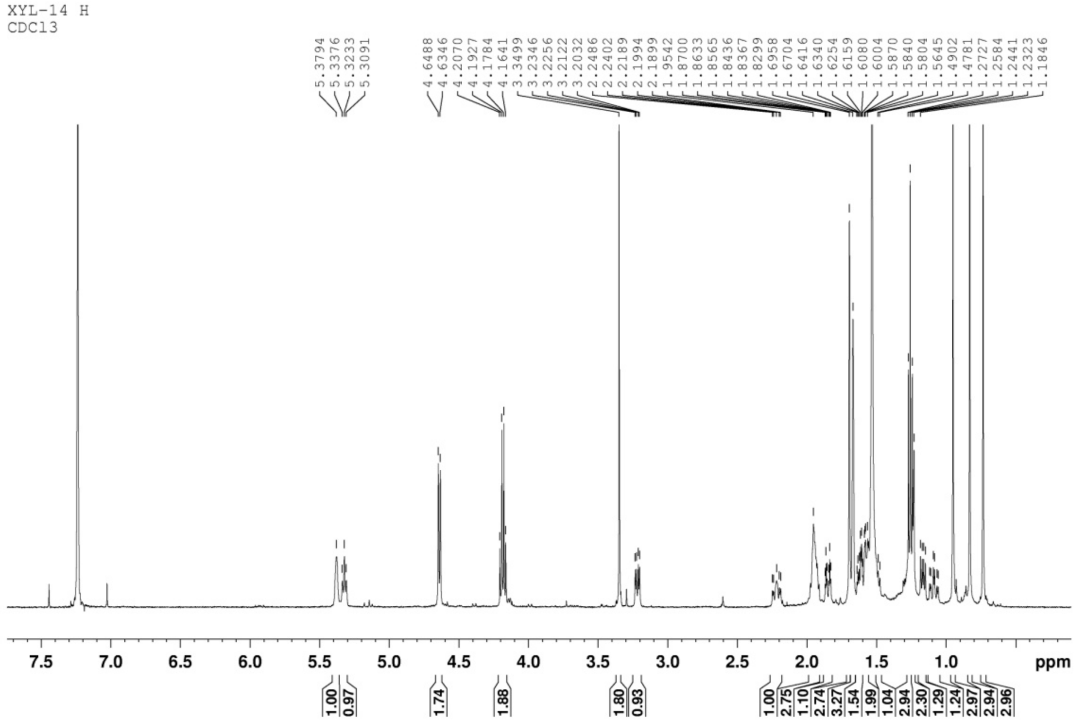

Figure S1. ^{1}H-NMR (500 MHz, $CDCl_3$) spectrum of compound **1**.

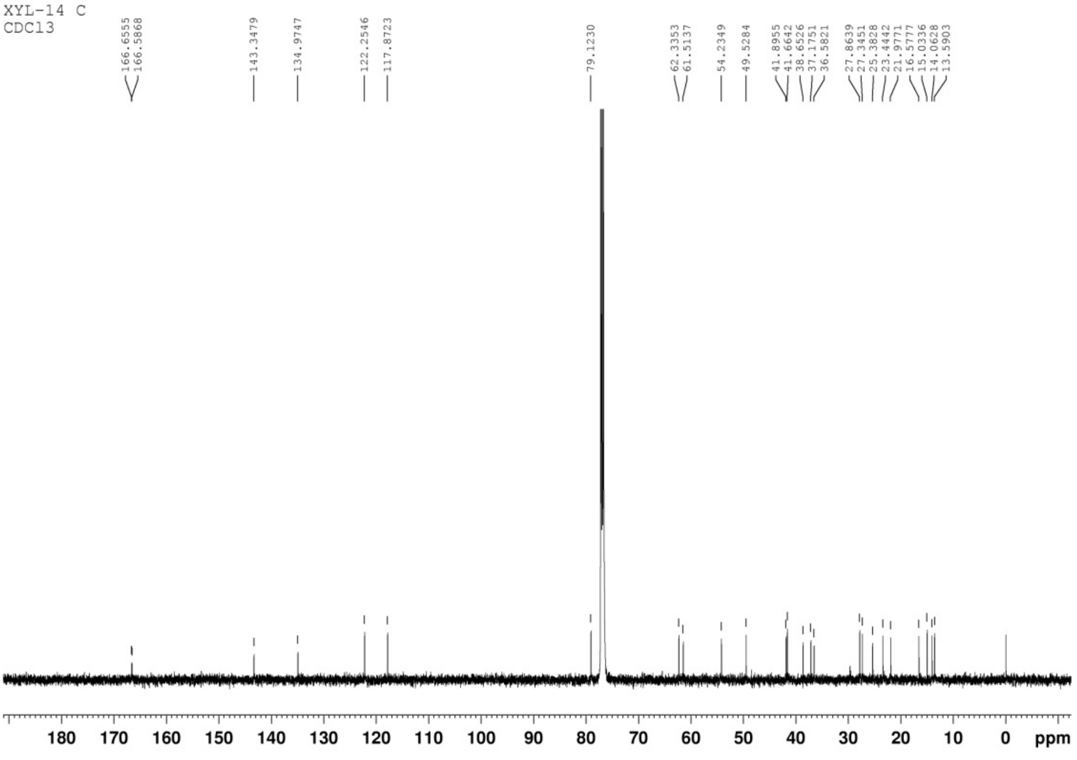

Figure S2. ^{13}C-NMR (125 MHz, $CDCl_3$) spectrum of compound **1**.

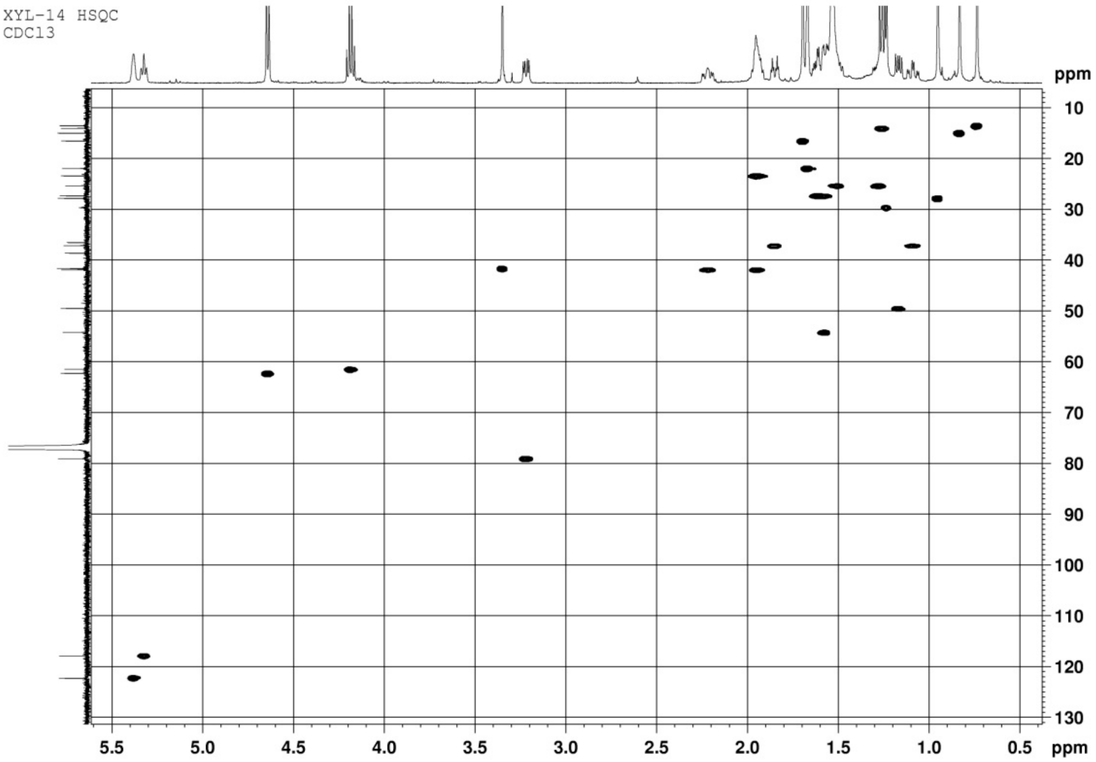

Figure S3. HSQC spectrum of compound **1**.

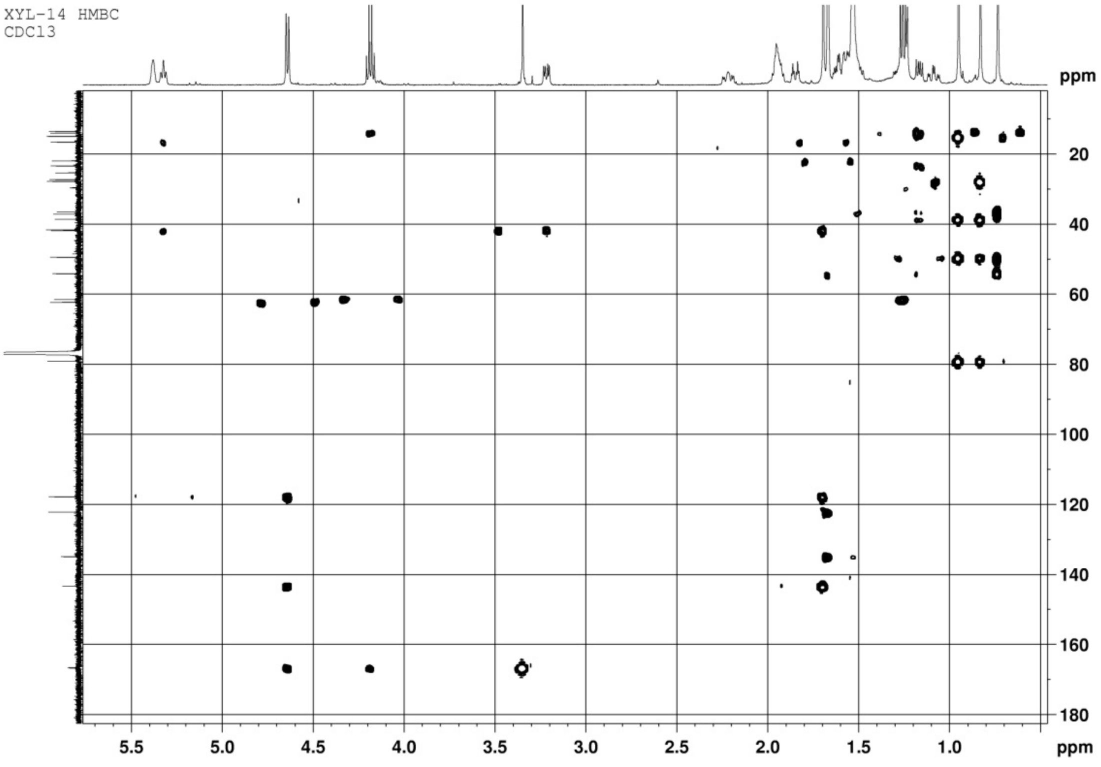

Figure S4. HMBC spectrum of compound **1**.

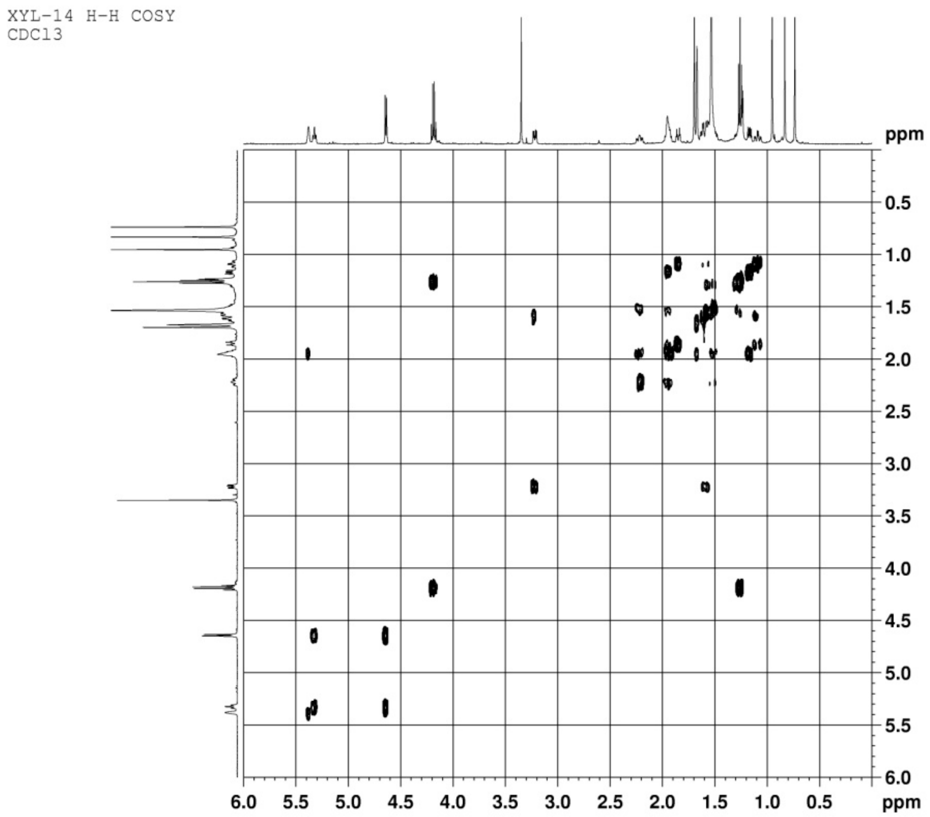

Figure S5. ^{1}H-^{1}H COSY spectrum of compound **1**.

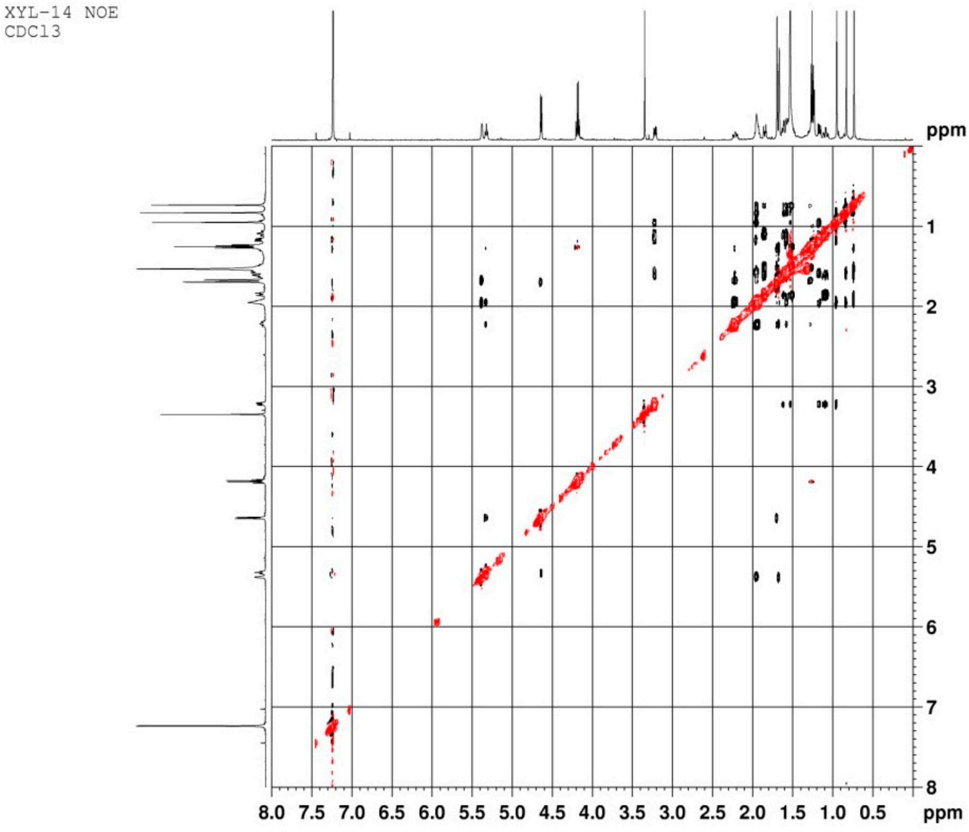

Figure S6. NOESY spectrum of compound **1**.

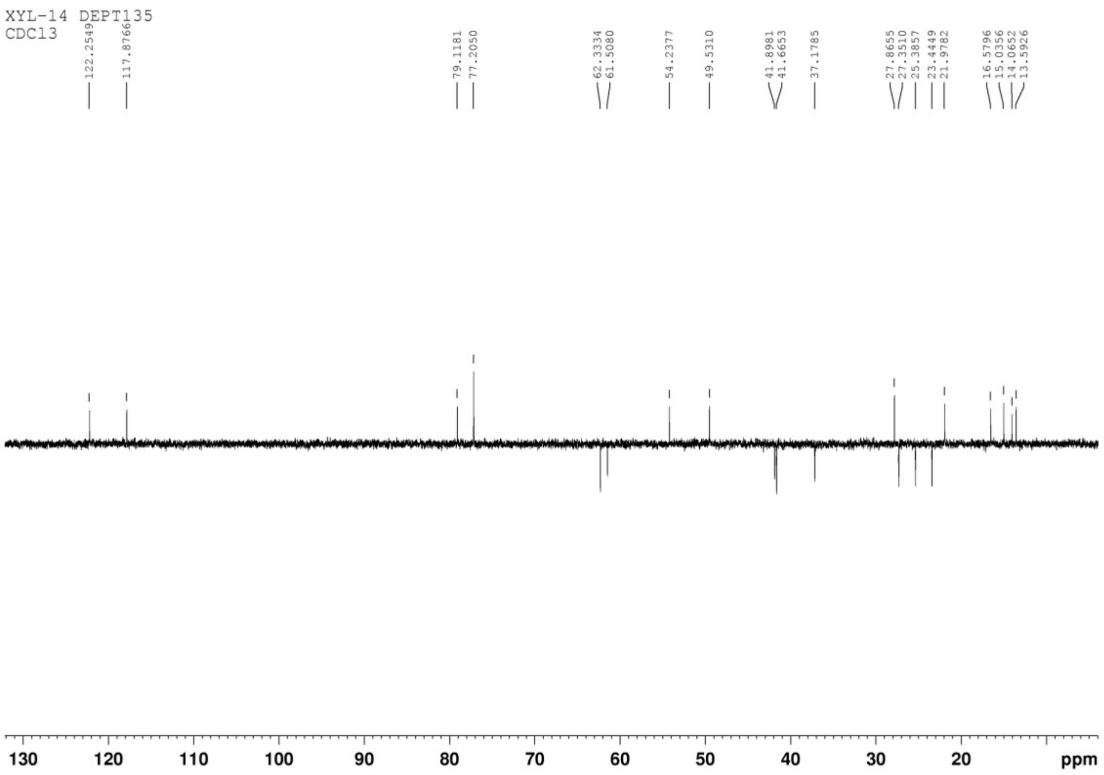

Figure S7. DEPT 135 spectrum of compound **1**.

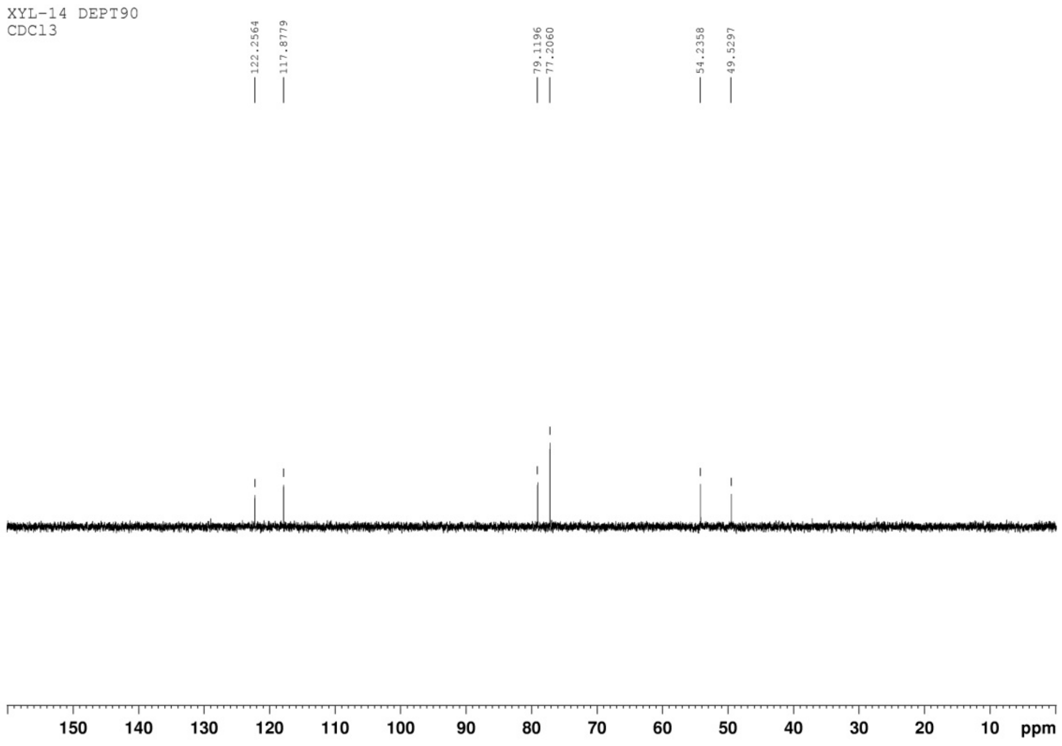

Figure S8. DEPT 90 spectrum of compound **1**.

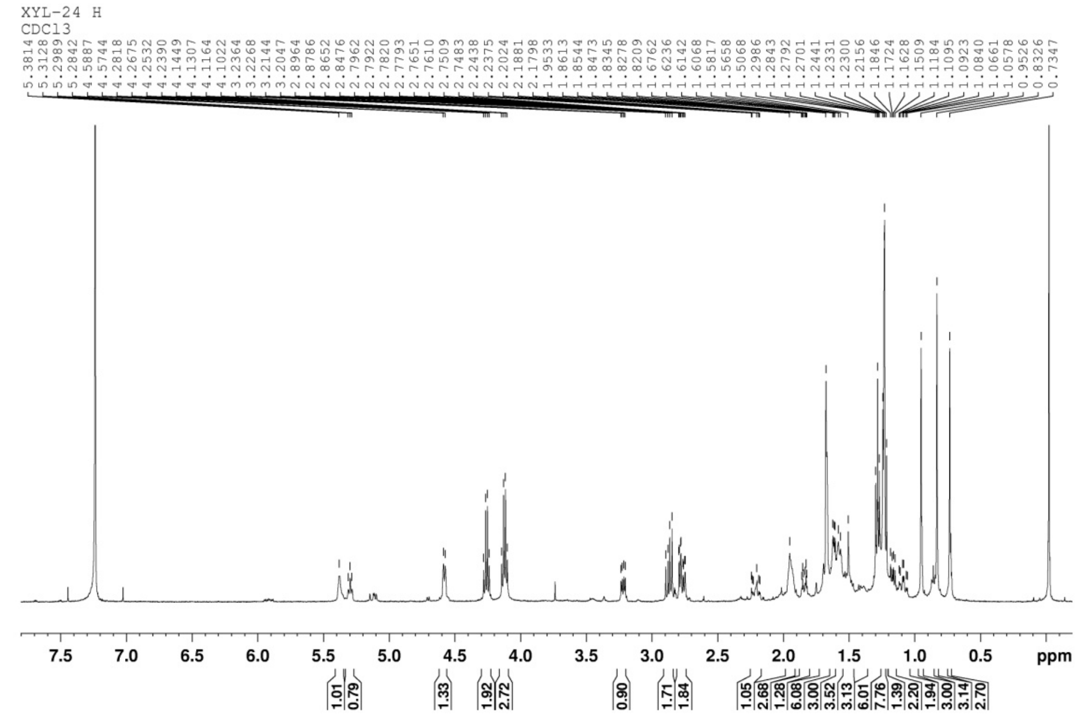

Figure S9. ^{1}H-NMR (500 MHz, $CDCl_3$) spectrum of compound **2**.

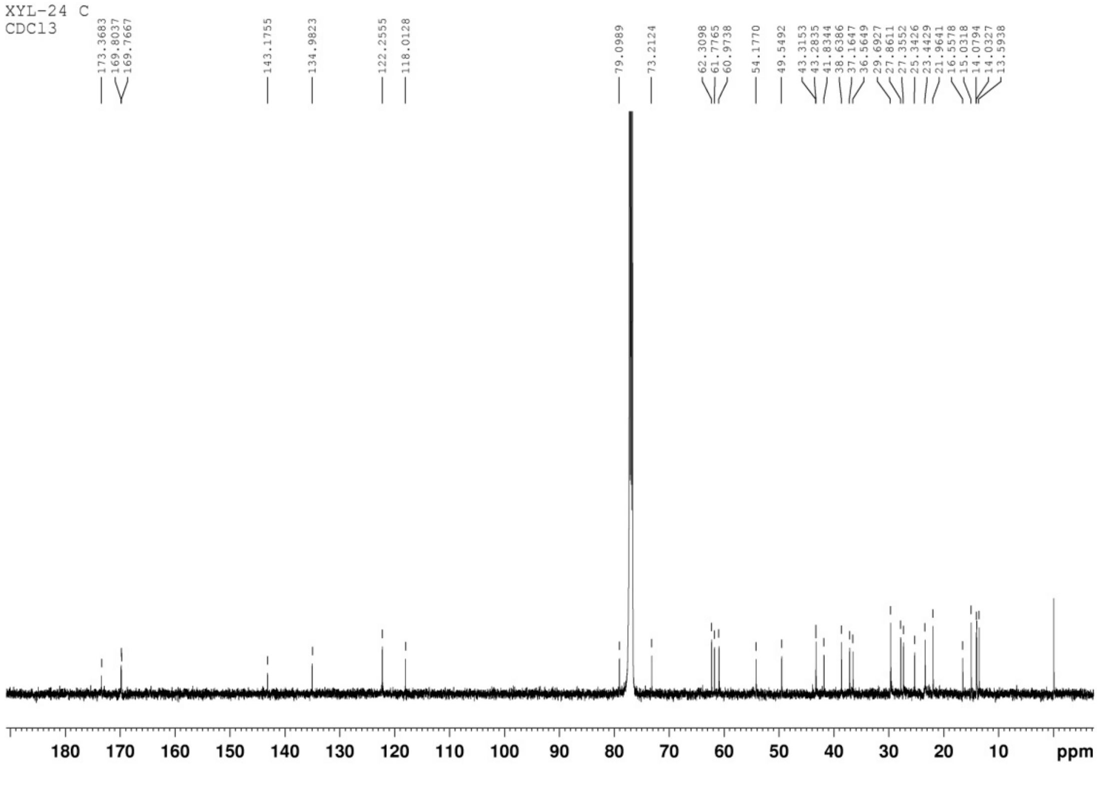

Figure S10. ^{13}C-NMR (125 MHz, $CDCl_3$) spectrum of compound **2**.

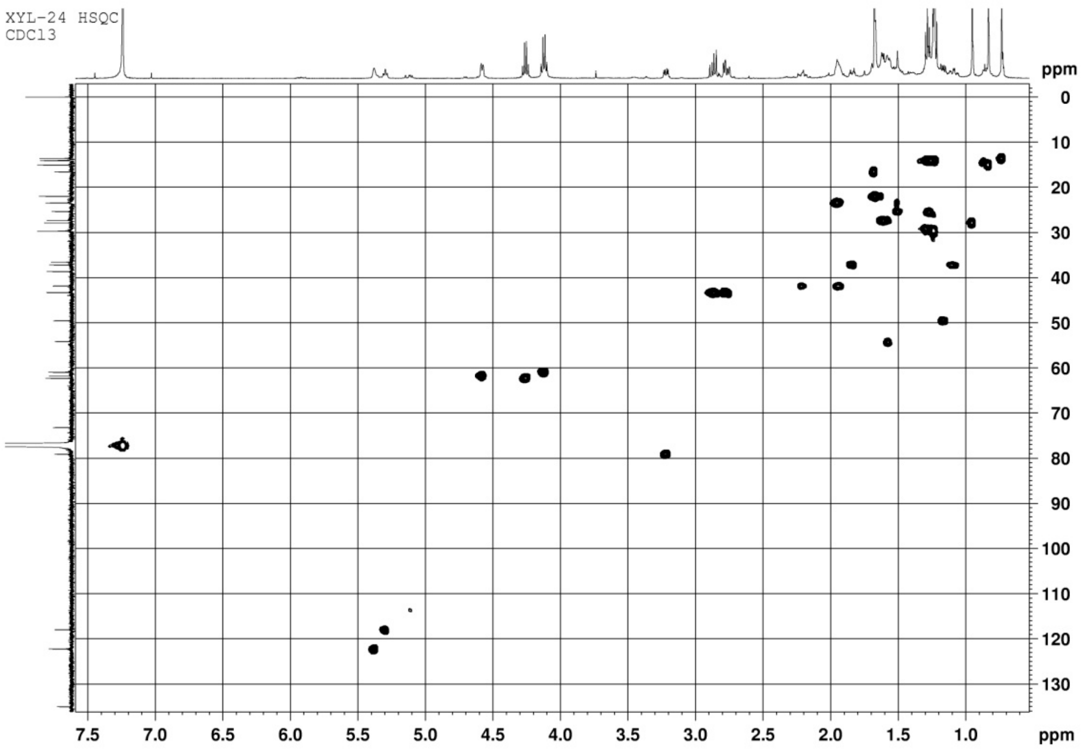

Figure S11. HSQC spectrum of compound **2**.

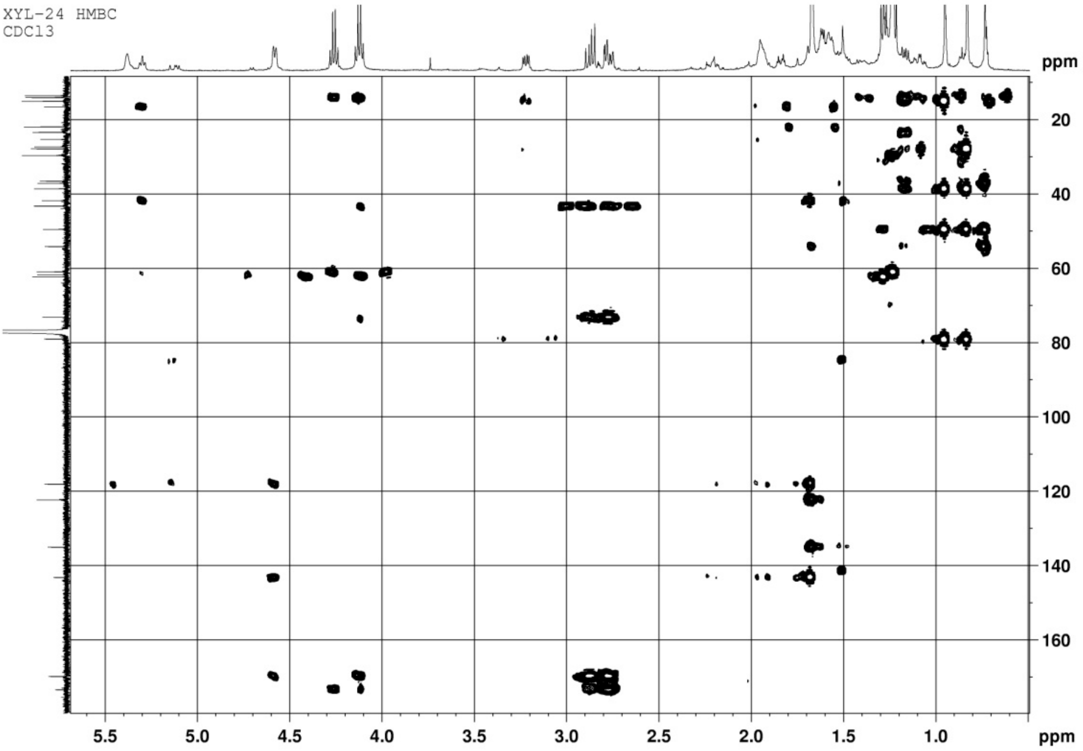

Figure S12. HMBC spectrum of compound **2**.

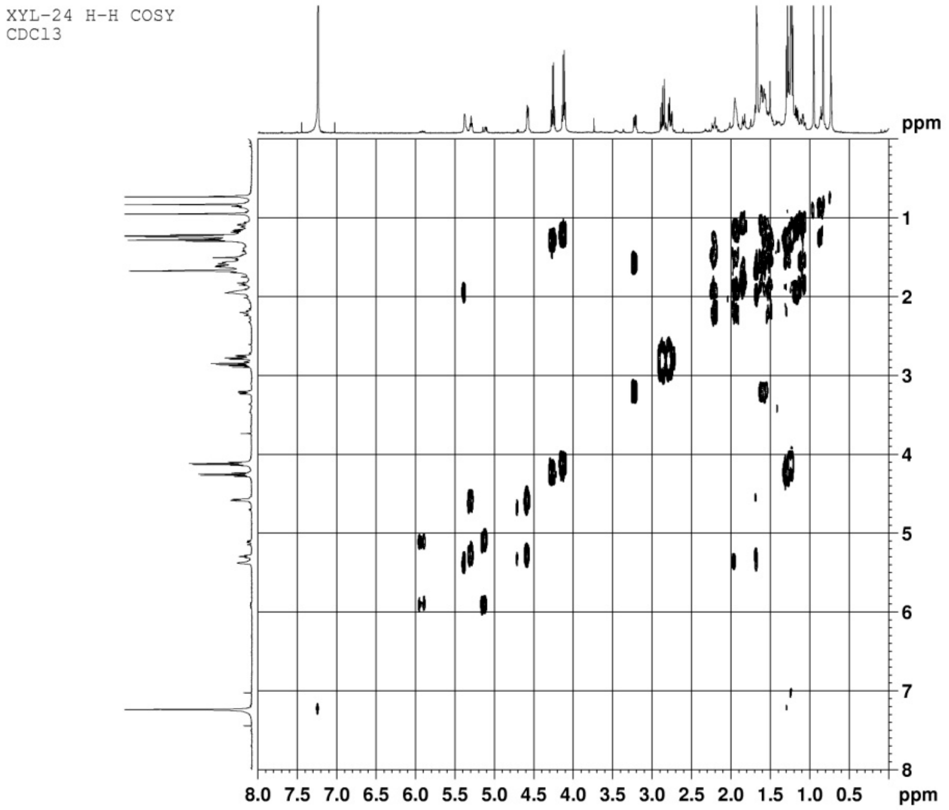

Figure S13. ^{1}H-^{1}H COSY spectrum of compound **2**.

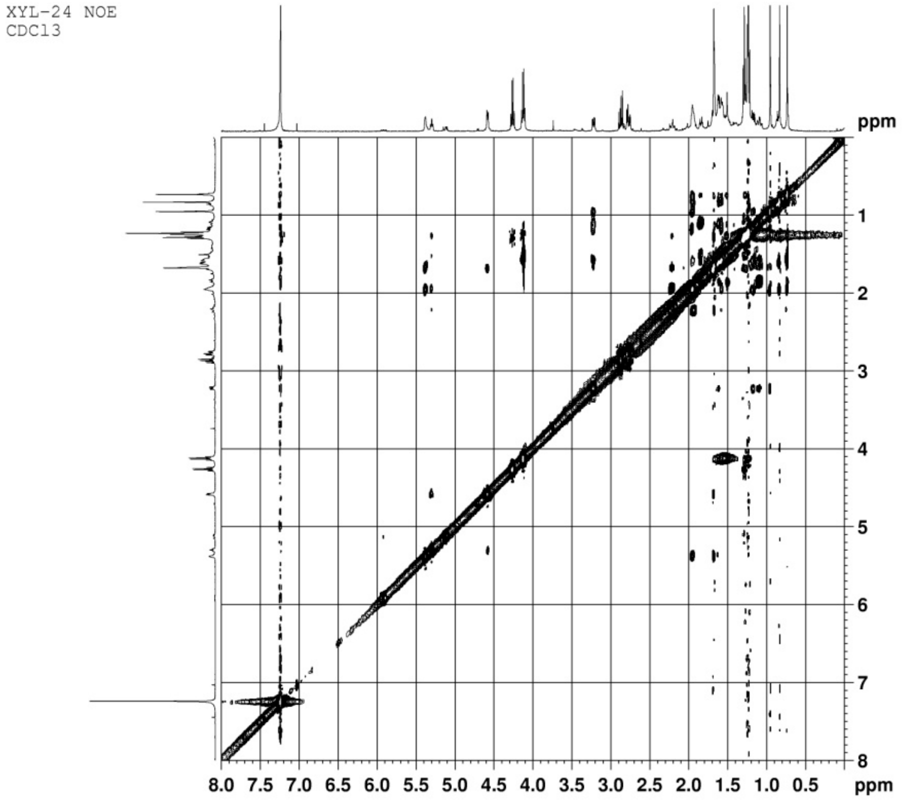

Figure S14. NOESY spectrum of compound **2**.

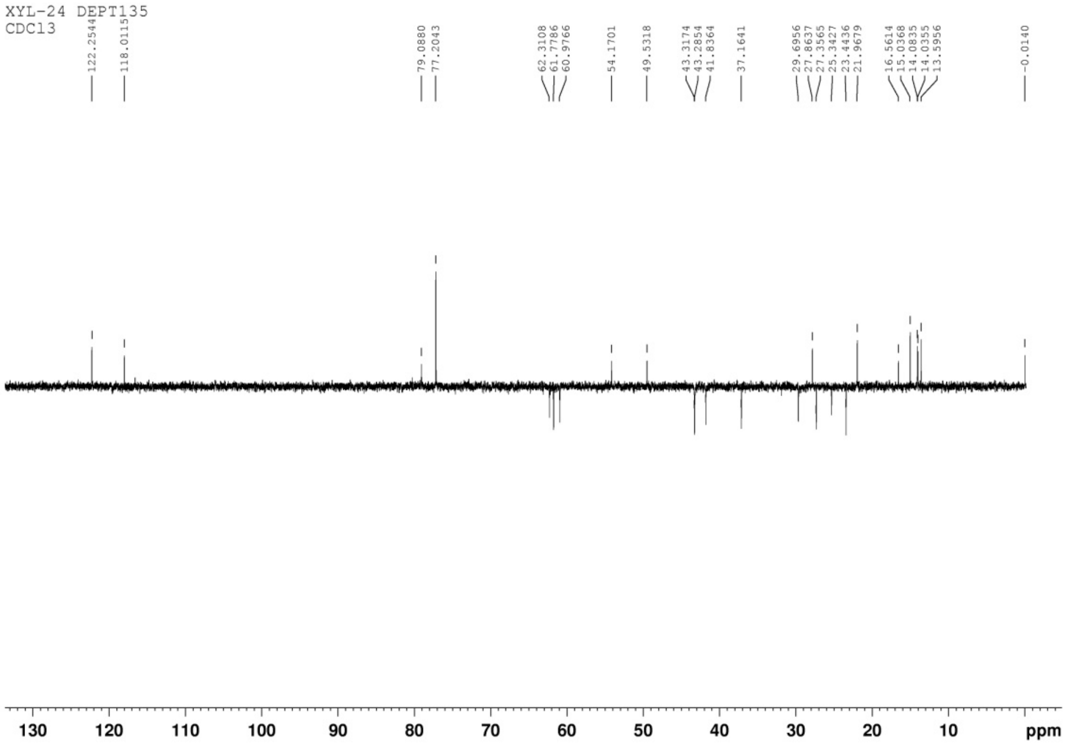

Figure S15. DEPT 135 spectrum of compound **2**.

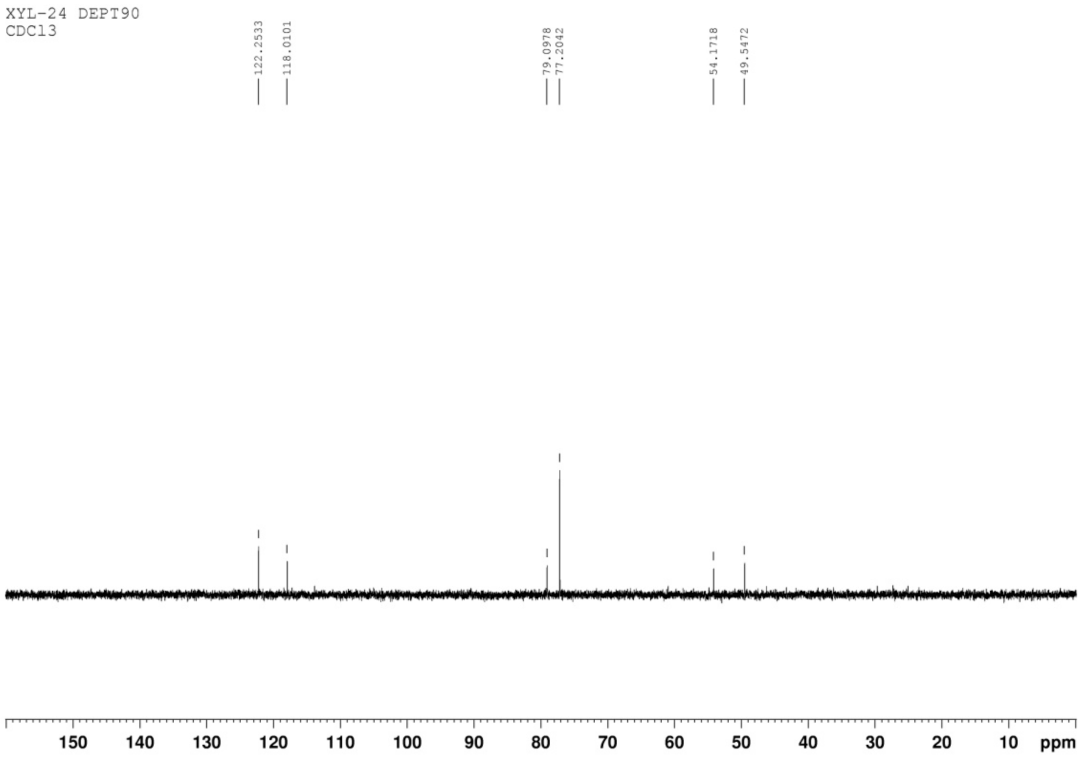

Figure S16. DEPT 90 spectrum of compound **2**.

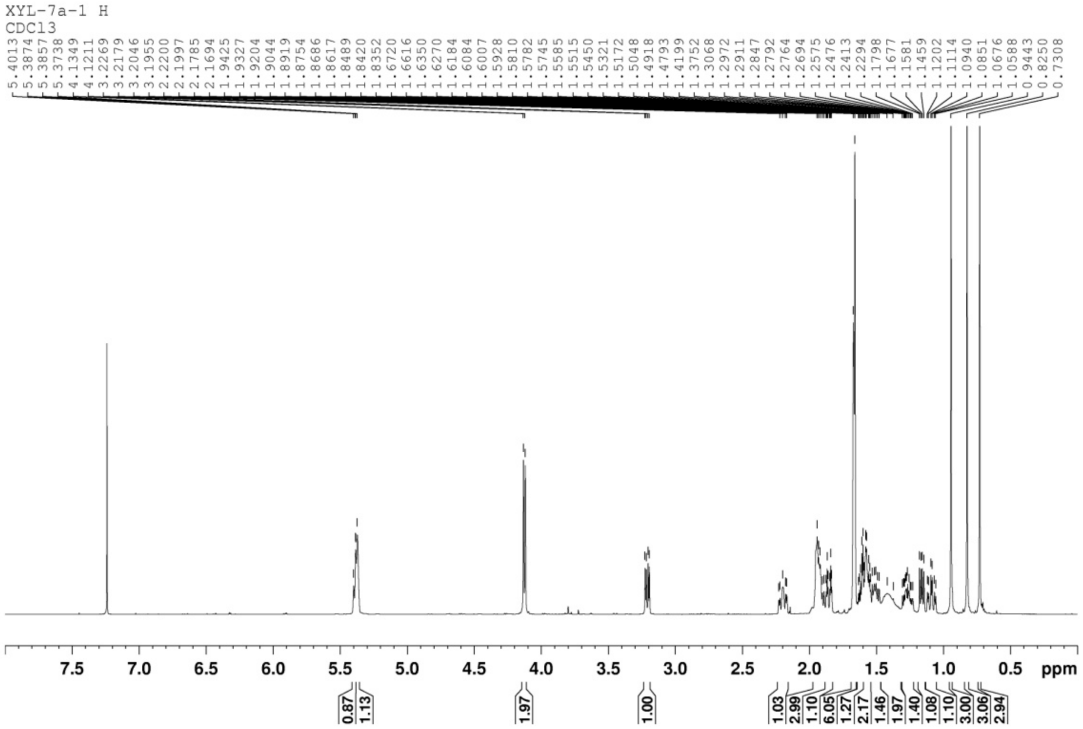

Figure S17. ^{1}H-NMR (500 MHz, $CDCl_3$) spectrum of compound **3**.

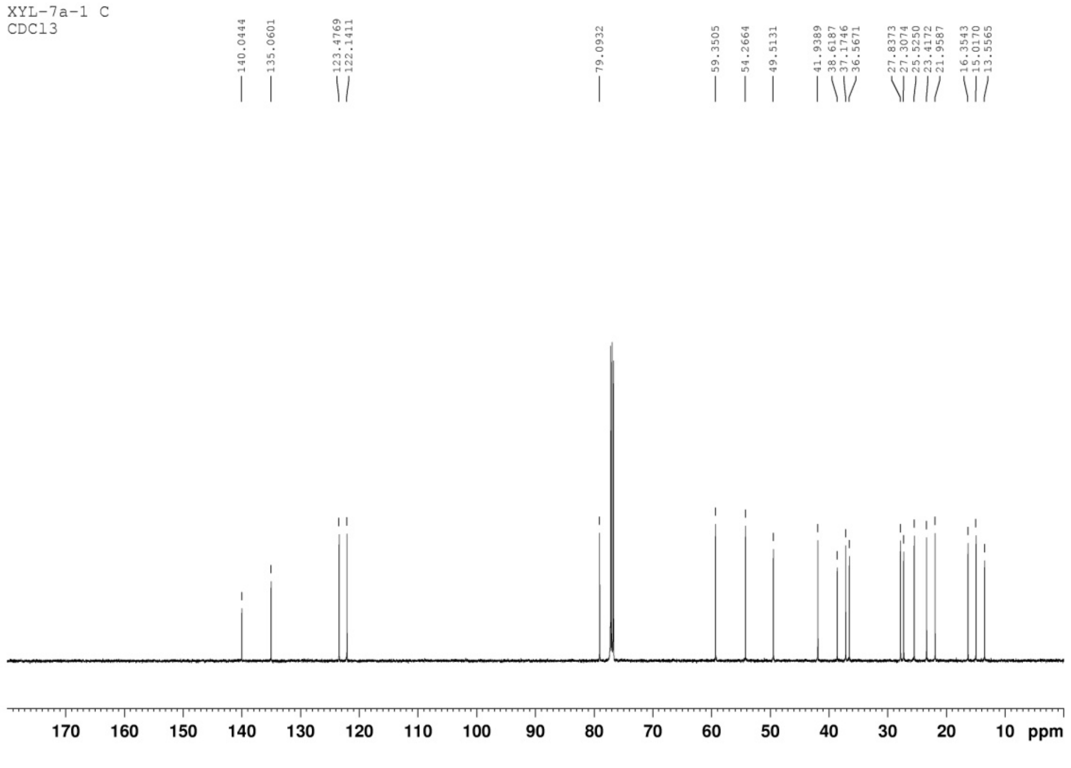

Figure S18. ^{13}C-NMR (125 MHz, $CDCl_3$) spectrum of compound **3**.

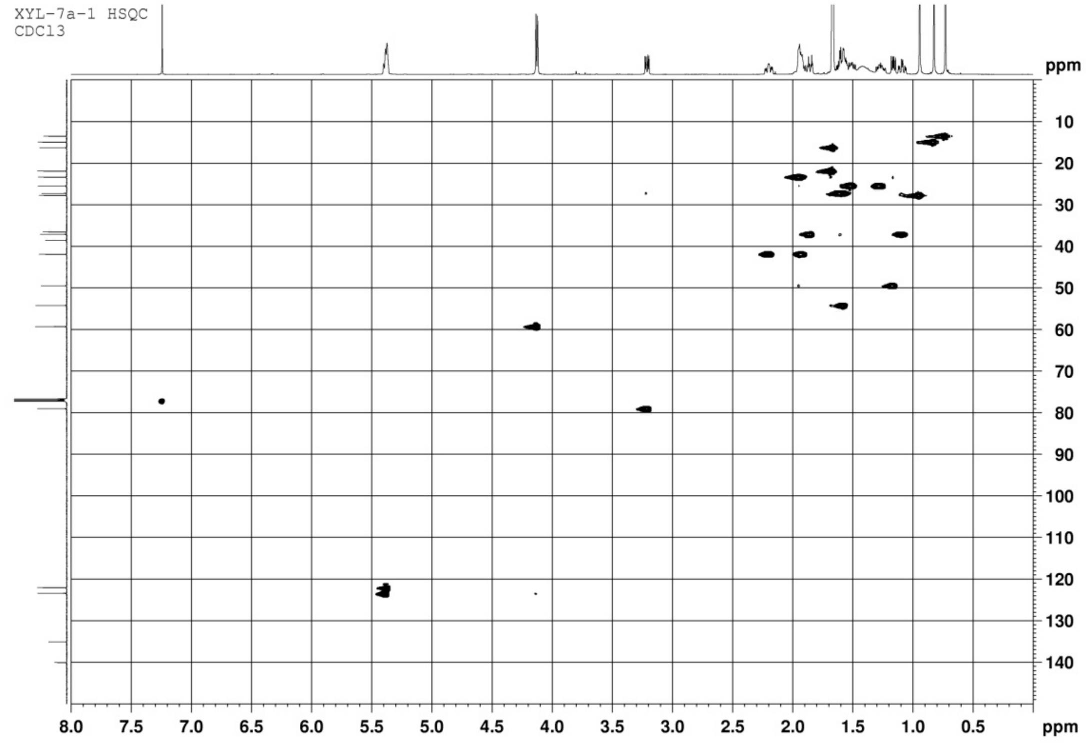

Figure S19. HSQC spectrum of compound **3**.

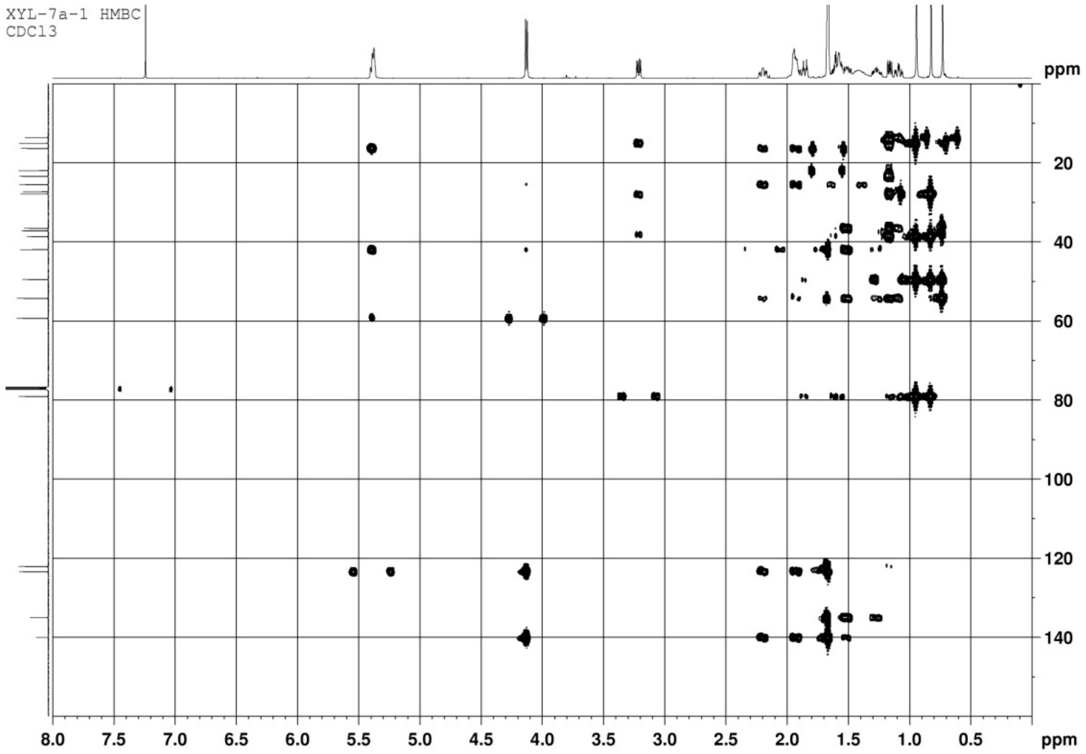

Figure S20. HMBC spectrum of compound **3**.

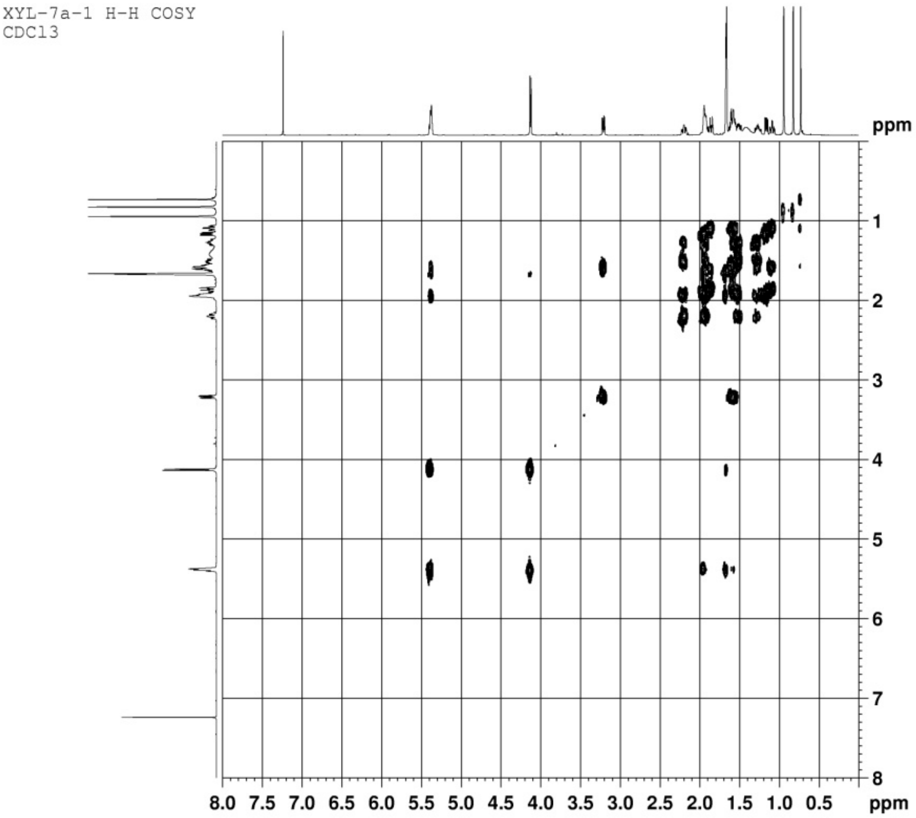

Figure S21. ^1H-^1HCOSY spectrum of compound **3**.

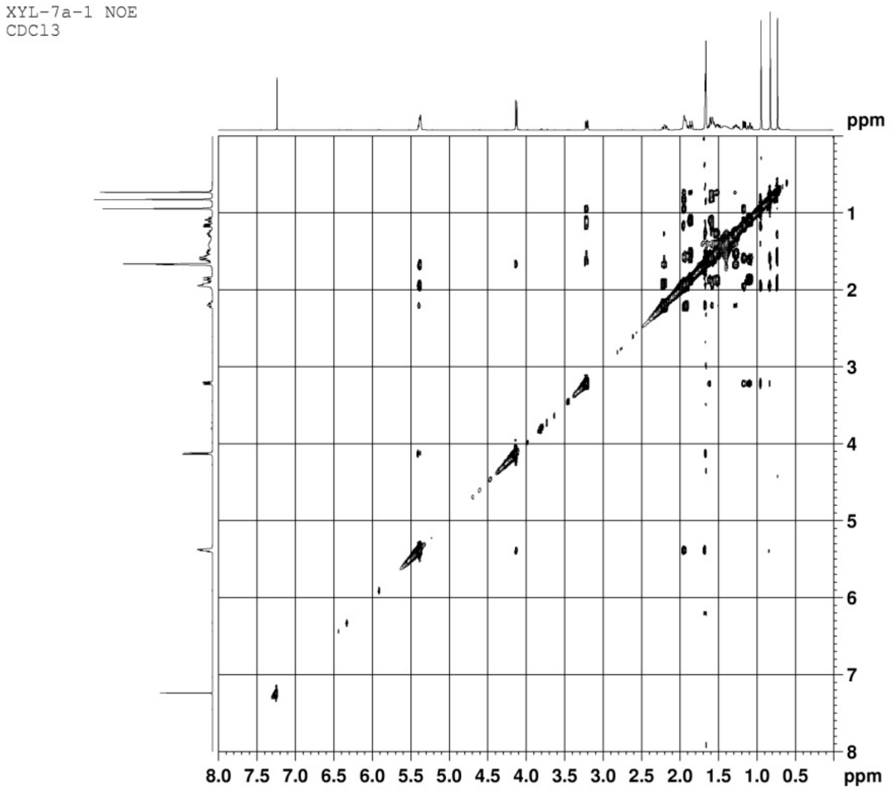

Figure S22. NOESY spectrum of compound **3**.

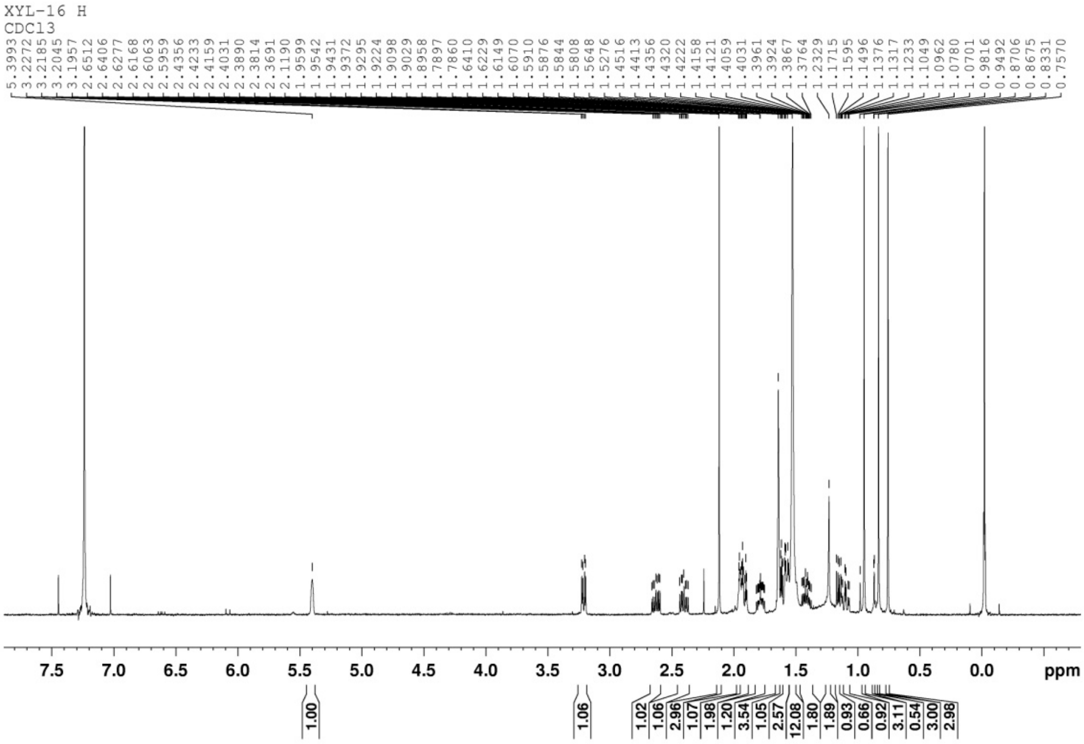

Figure S23. ^{1}H-NMR (500 MHz, $CDCl_3$) spectrum of compound **4**.

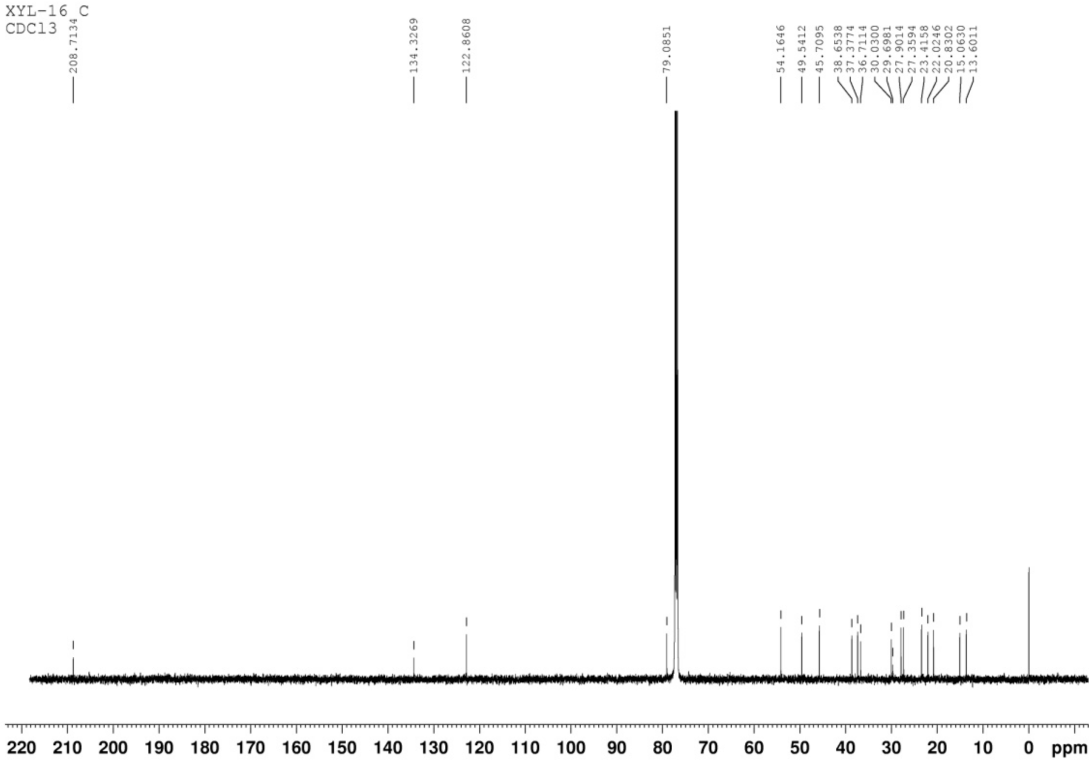

Figure S24. ^{13}C-NMR (125 MHz, $CDCl_3$) spectrum of compound **4**.

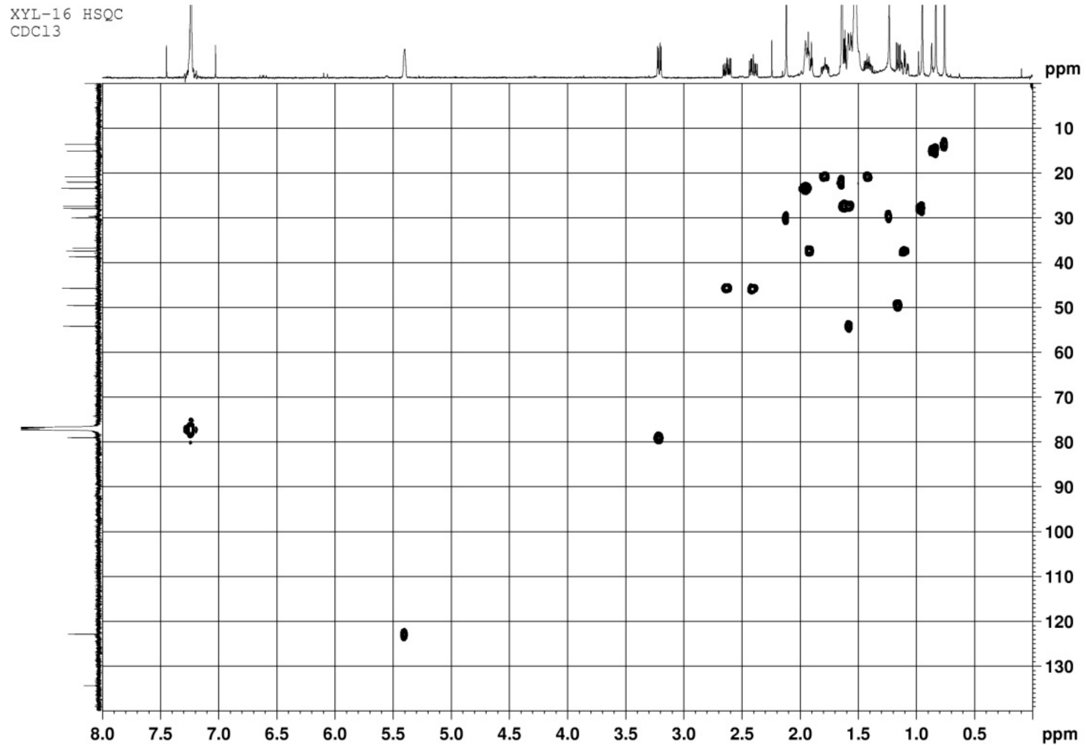

Figure S25. HSQC spectrum of compound **4**.

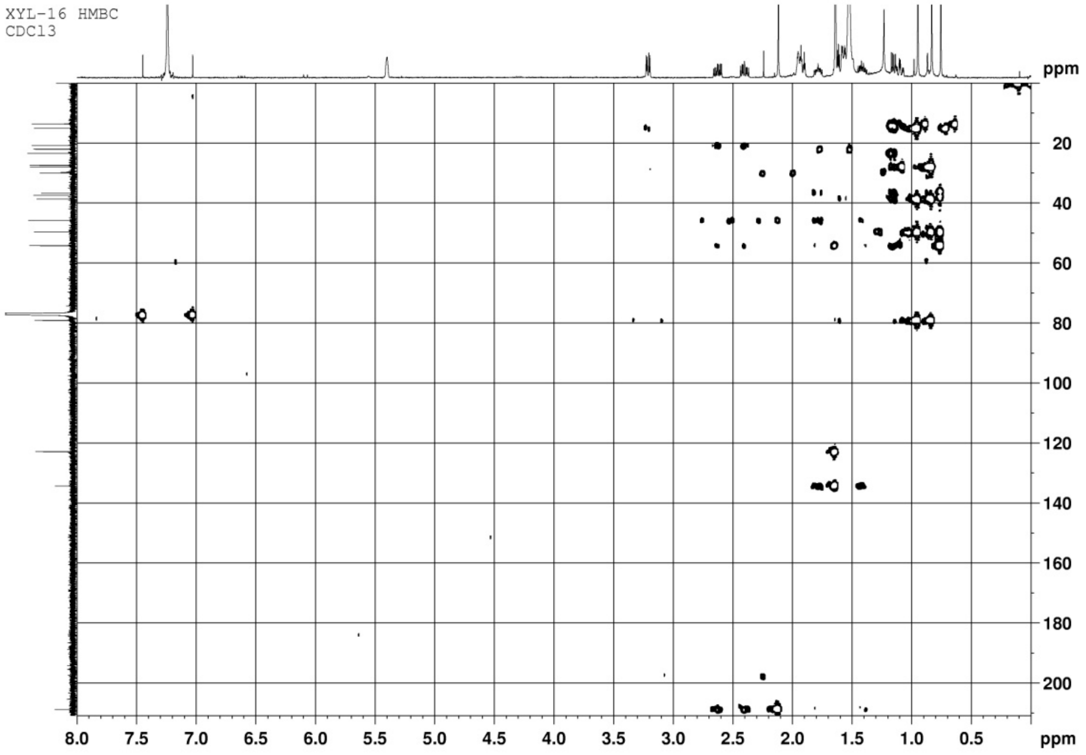

Figure S26. HMBC spectrum of compound **4**.

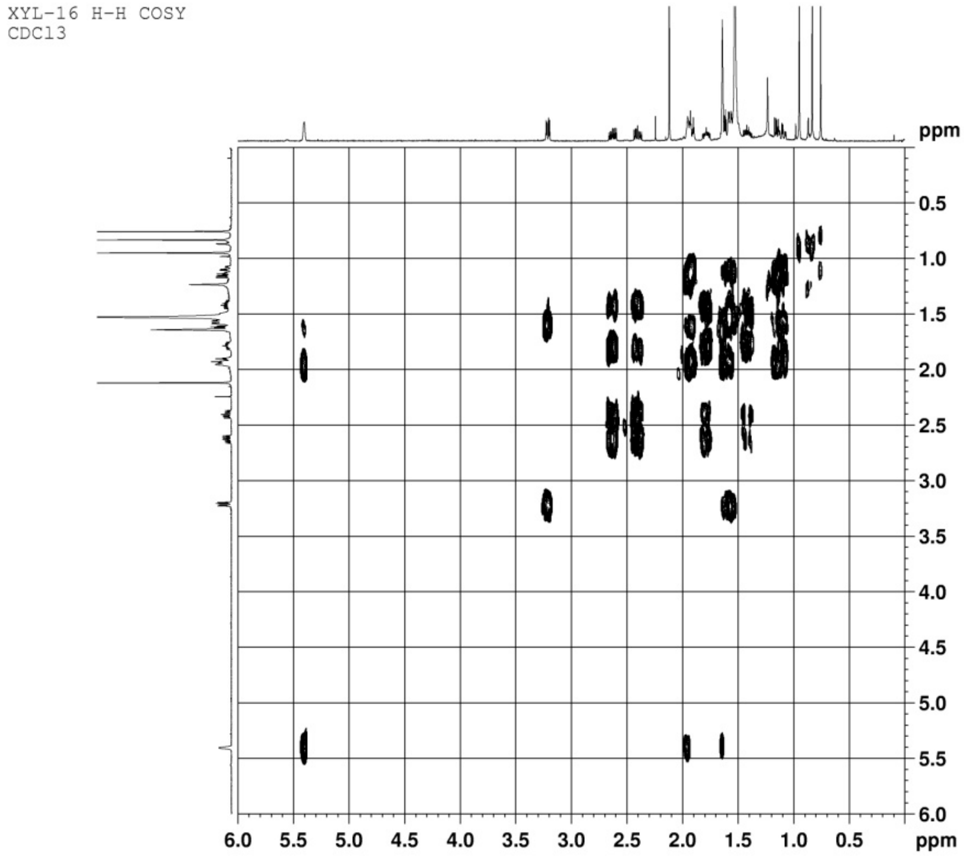

Figure S27. ^{1}H-^{1}HCOSY spectrum of compound **4**.

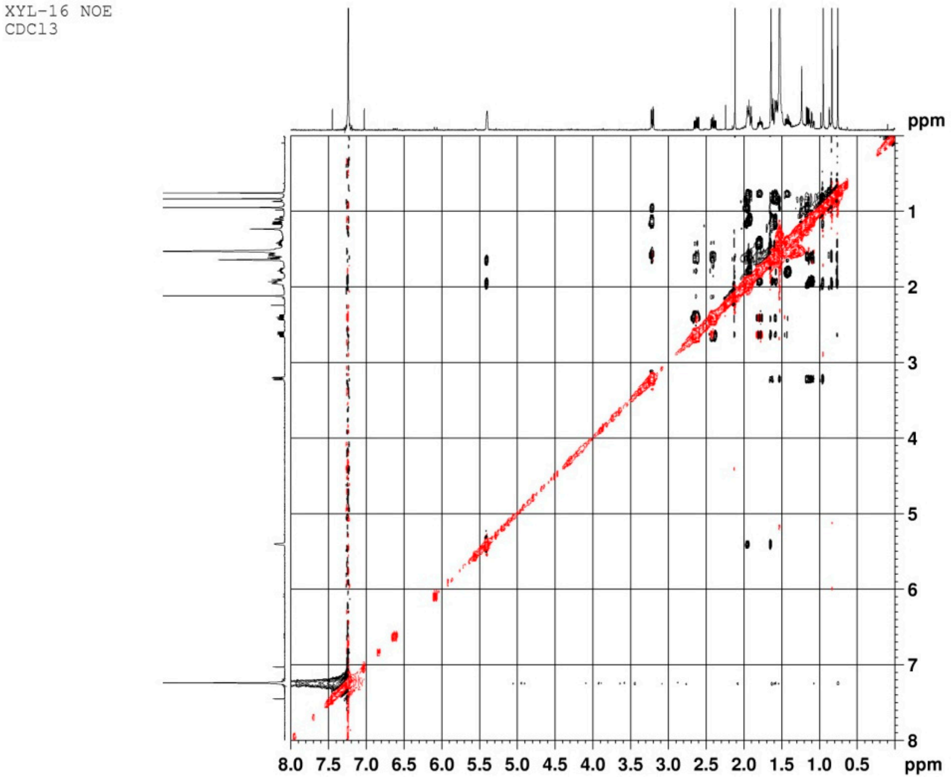

Figure S28. NOESY spectrum of compound **4**.
